# Supplementary material for: Comparison of triple-DMEK to pseudophakic-DMEK: A cohort study of 95 eyes
Source: PLoS One. 2022 May 12;17(5):e0267940. doi: 10.1371/journal.pone.0267940 (PMC9098022; doi:10.1371/journal.pone.0267940)
Supplement: S1 Table — (DOCX) [file pone.0267940.s001.docx]

S1 Table. Characteristics of the patient cohorts in the literature

| **Author (year) [Ref number]** | **Pseudophakic-DMEK *versus* triple DMEK group** | | | |
| --- | --- | --- | --- | --- |
|  | **No. Eyes (No. Patients)** | **Mean age, years (p value)** | **Female sex, %** | **FECD stage (p value)** |
| Chaurasia (2014) [29] | (292) *vs.* (200)^a^ | 70 *vs.* 64 (p<0.0001) | 58% *vs.* 61% | Conventional grading not performed; preoperative BSCVA 0.35 *vs.* 0.30 logMAR (p<0.0001) |
| Shahnazaryan (2020) [28] | 34 (29) *vs.* 80 (56) | 68 *vs.* 67 (p<0.0001) | 67% *vs.* 89% | Stages not described; preoperative BSCVA 0.47 *vs.* 0.56 (p<0.0001) |
| Ighani (2019) [30] | 8 (8) *vs.* 16 (15) | 73 for whole cohort | 75% for whole cohort | Stages not described; preoperative BSCVA 0.54 *vs.* 0.40 (p=0.09) |
| Our study | 55 (43) *vs.* 40 (34) | 72 *vs.* 70 (p=0.10) | 86% *vs.* 79% | Cornea guttata with corneal edema but not stromal scarring; preoperative BSCVA 0.50 *vs.* 0.50 (p=0.13) |

^a^ The number of patients was not specified
