# Supplementary material for: Comparison of triple-DMEK to pseudophakic-DMEK: A cohort study of 95 eyes
Source: PLoS One. 2022 May 12;17(5):e0267940. doi: 10.1371/journal.pone.0267940 (PMC9098022; doi:10.1371/journal.pone.0267940)
Supplement: Attachment — Submitted filename: Response to Reviewer.docx [file pone.0267940.s003.docx]

**Reply to Reviewer**

Reviewer #2: The authors have revised their manuscript according to the reviewers' suggestions.
However, a minor issue needs to be resolved:

The data shown in Table 1 does not correspond to the data about their own study (!) as shown in Supplemental Table S1.
Mean age of patients in the study is stated to be 70 (Triple DMEK) and 72 (Pseudophakic-DMEK) in table 1.
Supplemental Table S1 states "72 vs. 69".
Same for the number of patients (55(43) vs. 40(34) in table 1 and 55(43) vs. 34(40) in supplemental table S1).
Same for female sex (79% and 86% in table 1 versus 68% and 67% in supplemental table S1).
Same for BSCVA (0.50 in table 1 and 0.63/0.59 in supplemental table S1).

Actually, proofreading and noticing of such errors should be done by the authors themselves !

Reply: We sincerely apologize for this error and are grateful that you picked it up. The previous review identified errors in Table 1 that we corrected but then we overlooked correcting the data in Supplementary Table S1 as well. These corrections have been made, as shown below:

Supplementary Table S1. Characteristics of the patient cohorts in the literature

| **Author (year) [Ref number]** | **Pseudophakic-DMEK *versus* triple DMEK group** | | | |
| --- | --- | --- | --- | --- |
|  | **No. Eyes (No. Patients)** | **Mean age, years (p value)** | **Female sex, %** | **FECD stage (p value)** |
| Chaurasia (2014) [29] | (292) *vs.* (200)^a^ | 70 *vs.* 64 (p<0.0001) | 58% *vs.* 61% | Conventional grading not performed; preoperative BSCVA 0.35 *vs.* 0.30 logMAR (p<0.0001) |
| Shahnazaryan (2020) [28] | 34 (29) *vs.* 80 (56) | 68 *vs.* 67 (p<0.0001) | 67% *vs.* 89% | Stages not described; preoperative BSCVA 0.47 *vs.* 0.56 (p<0.0001) |
| Ighani (2019) [30] | 8 (8) *vs.* 16 (15) | 73 for whole cohort | 75% for whole cohort | Stages not described; preoperative BSCVA 0.54 *vs.* 0.40 (p=0.09) |
| Our study | 55 (43) *vs.* 40 (34) | 72 *vs.* 70 (p=0.10) | 86% *vs.* 79% | Cornea guttata with corneal edema but not stromal scarring; preoperative BSCVA 0.50 *vs.* 0.50 (p=0.13) |

^a^ The number of patients was not specified
